# Supplementary material for: Structured Framework and Genome Analysis of Magnaporthe grisea Inciting Pearl Millet Blast Disease Reveals Versatile Metabolic Pathways, Protein Families, and Virulence Factors
Source: J Fungi (Basel). 2022 Jun 9;8(6):614. doi: 10.3390/jof8060614 (PMC9225118; doi:10.3390/jof8060614)
Supplement: Supplementary file 1 [file jof-08-00614-s001.zip › jof-1644807-supplementary.pdf]

Supplementary file

Article

Structured framework and genome analysis of *Magnaporthe grisea* inciting pearl millet blast disease reveals versatile metabolic pathways, protein families, and virulence factors

Bhaskar Reddy[1*], Sahil Mehta[2,3], Ganesan Prakash[1], Neelam Sheoran[1], and Aundy Kumar[1*]

[1] Division of Plant Pathology, ICAR-Indian Agricultural Research Institute, New Delhi-110012, India; e-24breddy@gmail.com (B.R.); prakashg@iari.res.in (G.P.); haardikk@yahoo.co.in (N.S.); kumar@iari.res.in (A.K.)

[2] Crop Improvement Group, International Centre for Genetic Engineering and Biotechnology, New Delhi-110067, India; sahil.mehta@icgeb.res.in (S.M.)

[3] School of Agricultural Sciences, K. R. Mangalam University, Sohna Rural, Haryana–122103, India

* Correspondence: kumar@iari.res.in(AK); Orcid ID:https://orcid.org/0000-0002-7401-9885(AK);24breddy@gmail.com (BR); Orcid ID:https://orcid.org/0000-0002-8177-9305 (BR)

Citation: Lastname, F.; Lastname, F.; Lastname, F. Title. *J. Fungi* **2022**, *8*, 614. https://doi.org/10.3390/jof8060614

Academic Editor: FirstnameLastname

Received: date
Accepted: date
Published: date

Publisher's Note: MDPI stays neutral with regard to jurisdictional claims in published maps and institutional affiliations.

Copyright:© 2022by the authors. Submitted for possible open access publication under the terms and conditions of the Creative Commons Attribution (CC BY) license (https://creativecommons.org/licenses/by/4.0/).

Abstract: *Magnaporthe grisea* (T.T. Herbert) M.E. Barr is a major fungal phytopathogen that causes blast disease in the cereals resulting in economic losses worldwide. An in-depth understanding of the basis of virulence and ecological adaptation of *M. grisea* is vital for devising effective disease management strategies. Here, we aimed to determine the genomic basis of the pathogenicity and underlying biochemical pathways in *Magnaporthe* using the genome sequence of a pearl millet infecting *M. grisea* PMg_Dl generated by dual NGS techniques, Illumina NextSeq 500 and PacBio RS II. The short and long nucleotide reads could be draft assembled in 341 contigsshowed a genome size of 47.89 Mb with the N50 value of 765.4 Kb. *Magnaporthe grisea* PMg_Dl showed an average nucleotide identity (ANI) of 86 % and 98 % with *M. oryzae* and *Pyricularia pennisetigena*, respectively. The gene-calling method revealed a total of 10,218 genes and 10,184 protein-coding sequences in the genome of PMg_Dl. InterProScan of predicted protein showed a distinct 3637 protein families and 695 superfamilies in the PMg_Dl genome. *In silico* virulence-analysis revealed the presence of 51-VFs and 539-CAZymes in the genome. Genomic region for the biosynthesis of cellulolytic endo-glucanase and beta-glucosidase, as well as pectinolytic endo-polygalacturonase, pectin-esterase, and pectate-lyases (pectinolytic) were detected. Signaling pathways modulated by MAPK, PI3K-Akt, AMPK, and mTOR were also deciphered. Multicopy sequences suggestive of transposable elements such as Type LTR, LTR/Copia, LTR/Gypsy, DNA/TcMar-Fot1, and Type LINE were recorded. The genomic resource presented here will be of immense use in the development of molecular marker and diagnosis, population genetics, disease management, and molecular taxonomy, and also provide a genomic reference for ascomycetous genome investigations in the future.

Keywords: Blast disease, *Magnaporthe*, Sequencing, Genome assembly, Protein family, CAZymes, Virulence, Effectors

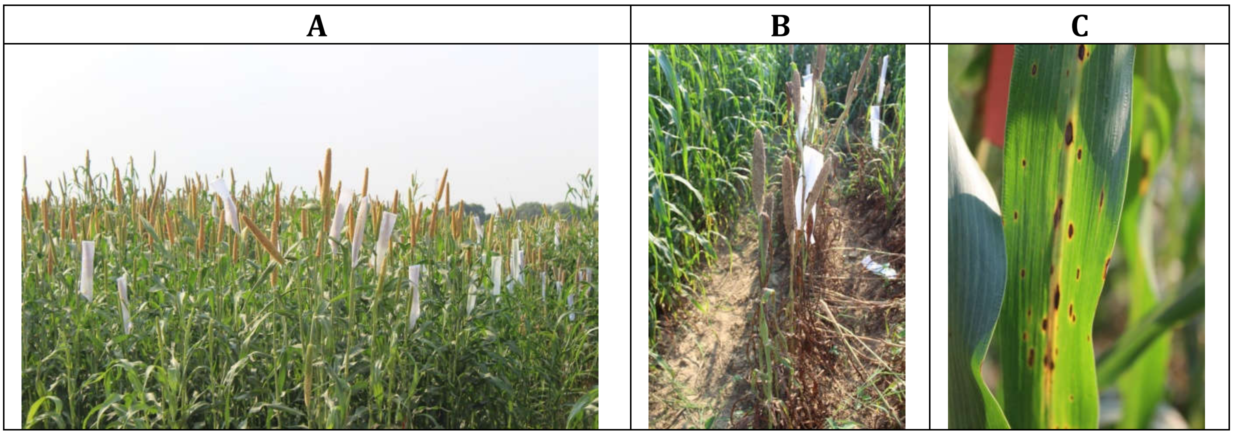

Figure S1. (A) View of pearl millet field at IARI Farm, New Delhi; **(B)**. Outbreak of blast incidence in pearl millet; **(C)**. Blast lesions on leaf of pearl millet.

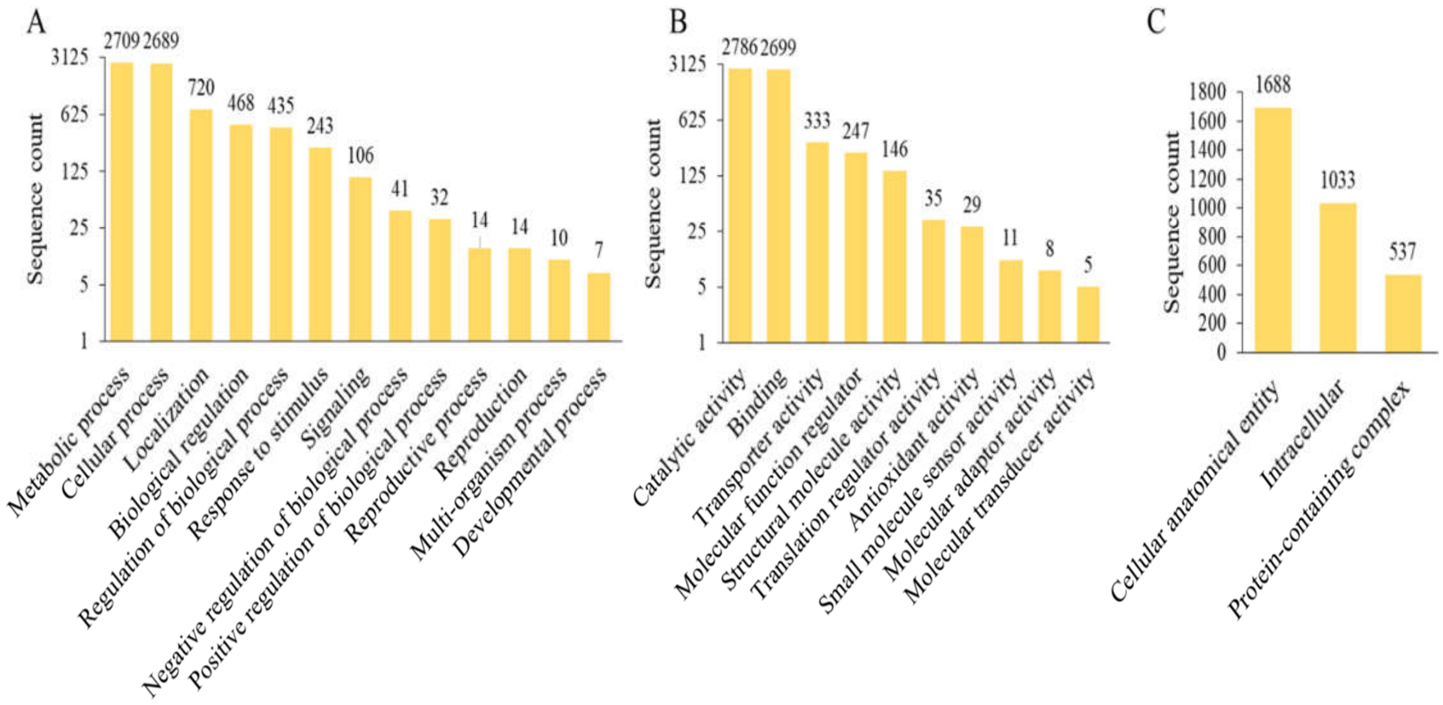

Figure S2. Functional annotation of predicted genes/proteins of *M. grisea* PMg_Dl in GO terms **(A)** Biological process-related GO groups, **(B)** Molecular function-related GO groups, and **(C)** Cellular component-related GO groups.
